# High-Radix Taylor-Optimized Tone Mapping Processor for Adaptive 4K HDR Video at 30 FPS

**DOI:** 10.3390/s25133887

**Published:** 2025-06-22

**Authors:** Xianglong Wang, Zhiyong Lai, Lei Chen, Fengwei An

**Affiliations:** 1 School of Microelectronics, Southern University of Science and Technology, Shenzhen 518055, China; 12031015@mail.sustech.edu.cn (X.W.); 12432952@mail.sustech.edu.cn (Z.L.); chenl33@sustech.edu.cn (L.C.); 2 State Key Laboratory of Quantum Functional Materials, Southern University of Science and Technology, Shenzhen 518055, China

**Keywords:** HDR, tone mapping, FPGA

## Abstract

High Dynamic Range (HDR) imaging is capable of capturing vivid and lifelike visual effects, which are crucial for fields such as computer vision, photography, and medical imaging. However, real-time processing of HDR content remains challenging due to the computational complexity of tone mapping algorithms and the inherent limitations of Low Dynamic Range (LDR) capture systems. This paper presents an adaptive HDR tone mapping processor that achieves high computational efficiency and robust image quality under varying exposure conditions. By integrating an exposure-adaptive factor into a bilateral filtering framework, we dynamically optimize parameters to achieve consistent performance across fluctuating illumination conditions. Further, we introduce a high-radix Taylor expansion technique to accelerate floating-point logarithmic and exponential operations, significantly reducing resource overhead while maintaining precision. The proposed architecture, implemented on a Xilinx XCVU9P FPGA, operates at 250 MHz and processes 4K video at 30 frames per second (FPS), outperforming state-of-the-art designs in both throughput and hardware efficiency. Experimental results demonstrate superior image fidelity with an average Tone Mapping Quality Index (TMQI): 0.9314 and 43% fewer logic resources compared to existing solutions, enabling real-time HDR processing for high-resolution applications.

## 1. Introduction

Tone mapping serves as a fundamental technique in computer graphics and vision, enabling the conversion of High Dynamic Range (HDR) images to Low Dynamic Range (LDR) formats for display on conventional monitors. While standard displays operate with 8-bit precision (0–255 dynamic range), natural scenes exhibit near-infinite dynamic ranges (0–+∞), necessitating HDR’s higher bit-depth representations (e.g., half/single-precision floating-point) to accurately capture real-world lighting. HDR imaging preserves critical details in challenging lighting conditions, such as backlit scenes with simultaneous highlight and shadow retention. Consequently, effective HDR-to-LDR conversion through tone mapping is essential for optimal visual fidelity on mainstream displays, establishing its pivotal role in modern computer vision systems.

Traditional tone mapping methodologies have evolved into two primary categories over decades of development. Global operators apply uniform mapping functions across entire images, offering computational simplicity through techniques like linear/logarithmic scaling, histogram equalization, and Reinhard’s method [1], albeit at the cost of compromised detail preservation. Conversely, local operators such as the Durand–Dorsey [2], Fattal [3], Drago [4], and Mantiuk [5] algorithms adapt to regional image characteristics, enhancing contrast and detail retention through spatially-variant processing. However, this improved visual quality incurs substantial computational overhead, limiting real-time applicability.

Recent advances in deep learning have revolutionized HDR tone mapping through data-driven approaches. Liang et al. [6] mitigated halo artifacts via hybrid ℓ1-ℓ0 decomposition, while Su et al. [7] achieved photorealistic outputs through their ETMO framework. Rana et al. [8] leveraged conditional GANs in DeepTMO to generate high-resolution mappings, and Yang et al. [9] unified illumination adaptation with noise suppression in LA-Net. Zhu et al. [10] further advanced structural preservation through diffusion models, demonstrating deep learning’s potential in complex tone mapping scenarios.

Significant advancements have also been made in real-time tone mapping implementations. Popović et al. [11] developed a pipeline architecture utilizing polynomial approximation to adaptively adjust pixel values, preserving high-contrast regions while slashing processing latency. Nosko et al. [12], combined with an innovative de-ghosting method and a local tone mapping operator, a breakthrough has been achieved in hardware efficiency and real-time performance. Ambalathankandy et al. [13] demonstrated a FPGA-validated a global–local adaptive algorithm leveraging localized histogram equalization for rapid tone compression. Yang et al. [14] introduced a direct bitstream processing method for wide dynamic range (WDR) sensors, deriving fine-grained histograms from mantissa-exponent statistical analysis to enable precision tone mapping. Meanwhile, Muneer et al. [15] proposed the HART operator, integrating histogram-based compression with human visual system (HVS) sensitivity modeling to optimize perceptual quality. Complementing these efforts, Kashyap et al. [16] implemented a resource-efficient logarithmic number system (LNS) via digital recursion, streamlining high-bit-width arithmetic and adaptive parameter optimization. Their resource reuse strategy further curtailed hardware overhead, achieving a 43% reduction in LUT usage compared to conventional designs.

Despite notable advancements in both deep learning and traditional tone mapping methodologies, the computational complexity inherent to nonlinear transformations persists as a critical barrier. While these operations are indispensable for achieving perceptually accurate mappings, their intensive resource requirements impose critical bottlenecks in real-time, high-resolution applications. Specifically, the dual demands of maintaining system throughput (>30 FPS for 4K streams) and sub-0.1 dB precision under escalating resolution standards (e.g., 8K/120 Hz) create an inversely proportional relationship between computational efficiency and output quality. Current architectures struggle to reconcile these competing priorities, with benchmark studies revealing up to 62% throughput degradation when processing Ultra-High-Definition (UHD) content compared to High-Definition (HD) equivalents [17]. This fundamental tension between algorithmic fidelity and real-time feasibility underscores the urgent need for hardware–algorithm co-optimization strategies.

The computational intensity of nonlinear tone mapping operations poses a fundamental challenge to real-time system implementation, particularly when processing high-resolution content (4K/8K) at video rates exceeding 60 FPS. Modern applications—including immersive Virtual Reality (VR) systems and UHD broadcast pipelines—require sub-frame latency (<16 ms for 60 Hz systems) while maintaining PSNR fidelity above 45 dB. Conventional software-based implementations struggle with these dual constraints, exhibiting exponential increases in cycle consumption (∝*N*^2^ for *N* × *N* kernels) that degrade throughput by 58–72% when scaling from HD to 4K resolutions. This performance gap necessitates novel architectural paradigms by hardware–algorithm co-design for resolution-independent complexity.

Equally critical is the pursuit of resource-efficient implementations that reconcile precision requirements with physical constraints. While dedicated accelerators using 28 nm FPGA or ASIC platforms can achieve 2.8 TOPS/W efficiency, their area costs escalate by 3–5 × compared to linear operators—a critical limitation for edge devices where the die area directly correlates with deployment feasibility. The power–area–product (PAP) metric reveals an acute tradeoff: implementations optimizing for <1.5 W power budgets typically sacrifice 0.5–1.5 dB in Peak Signal-to-Noise Ratio (PSNR) performance, while precision-oriented designs (<0.05 dB loss) consume 3.2–4.8 × more silicon resources [18]. This interdependence mandates co-optimization across all system layers, from algorithm approximation (e.g., 16-bit logarithmic quantization) to microarchitectural innovation (e.g., stochastic computation models).

The persistent challenges in computational intensity and resource management underscore the need for heterogeneous acceleration frameworks. Contemporary solutions combining approximate computing paradigms with precision-gated execution units demonstrate promising tradeoffs—achieving 40% power reduction versus conventional designs while maintaining a Structural Similarity Index (SSIM) > 0.98. However, true scalability requires fundamental rethinking of nonlinear function implementation, particularly for transcendental operations dominating 68–82% of tone mapping cycles. Emerging approaches leveraging high-radix polynomial expansions (order 8–12) coupled with dynamic precision scaling show particular promise, reducing LUT utilization by 35% compared to traditional Taylor-series implementations without compromising HVS-aligned quality metrics.

Despite advancements in tone mapping architectures, the computational complexity of nonlinear transformations persists as a critical bottleneck. Both deep learning and traditional methods exhibit exorbitant computational demands—particularly in real-time 4K/8K processing scenarios—where system throughput (>30 FPS) and sub-0.5 dB accuracy impose exacting demands. This complexity escalates implementation challenges and constrains real-time capabilities, necessitating architectural innovations. Furthermore, specialized hardware for high-precision nonlinear operations introduces critical power–area tradeoffs, often straining compact system designs. Optimal solutions require co-optimized hardware–algorithm frameworks that strategically balance precision against power, area, and throughput constraints.

Contributions: In this paper, we propose an adaptive and efficient HDR tone mapping processor designed for high-quality real-time processing of high-resolution, high-frame-rate images under varying exposure conditions.

Adaptive Parameter Adjustment: An exposure-adaptive computation method dynamically adjusts tone mapping parameters based on input image characteristics, ensuring stable results even for video streams with fluctuating exposures.

Hybrid Precision Architecture: A pixel-level pipeline architecture balances computational precision and resource efficiency by combining fixed-point arithmetic (for core operations) with floating-point units (reserved for high-precision tasks such as logarithmic/exponential functions). Bilateral filtering is optimized via lookup tables (LUTs) and approximate computations, reducing resource usage while maintaining accuracy.

Transcendental Function Acceleration: High-radix Taylor expansions accelerate floating-point natural logarithm and exponential operations, addressing computational bottlenecks in transcendental functions. The system achieves real-time 4K video processing at 30 FPS.

Implemented on the Xilinx XCVU9P FPGA platform, the design demonstrates competitive advantages in both performance and hardware resource efficiency.

The remainder of this paper is organized as follows. Section 2 details the algorithm process. Section 3 introduces the hardware architecture implementation. Section 4 presents the experimental results and compares them with other advanced works. Section 5 concludes the paper and suggests possible directions for future research.

## 2. Algorithm

### 2.1. Algorithm Workflow

**Figure 1:**
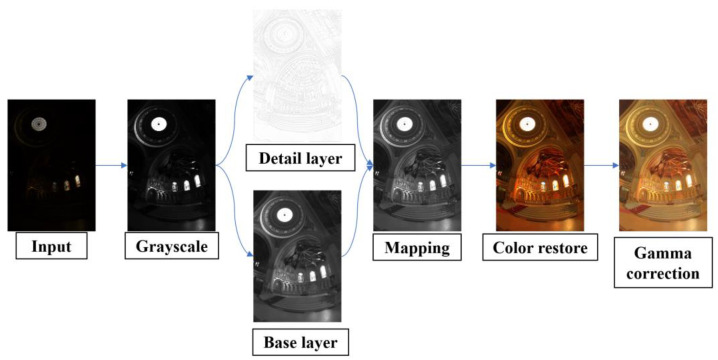
Workflow of our tone mapping processing.

Figure 1 demonstrates the complete processing workflow and stage-wise outcomes using the “memorial church” HDR image [19]—a validated benchmark in tone mapping research. This canonical example effectively illustrates the architecture’s response to extreme luminance variations (0.01–12,000 cd/m^2^), particularly in preserving stained glass details while compressing highlight regions. The algorithm process is shown in Algorithm 1.
**Algorithm 1 Tone mapping Algorithm with Updated Gamma Correction**1:**Input**: *img* (hdr format)2:**Output**: *outt*3:Initialization:4:  *height* = Get number of rows in the *img*5:  *width* = Get number of columns in the *img*6:  ϵ: 10^−6^7:Parameter:8:  *space_sigma* σₛ = min(*width*, *height*)/169:  $\mathrm{range}\_\mathrm{sigma}\sigma_{r}\;=\;(\ln(\max(\mathrm{intensity}))\;-\;\ln(\epsilon$))/10
10:  γ = ${\left(\frac{ln(2+mean(intensity))}{ln(3)}\right)}^{2}$11:  gamma = 0.512:Image Preprocessing:13:  *img:* Normalize image to [0, 1] range (*img = img/img.max*)14:Grayscale Conversion and Logarithmic Transformation:15: - Convert img to grayscale:16:  $intensity\;=\;0.299\cdot {img}_{R}+0.587\cdot {img}_{G}+0.114\cdot {img}_{B}+\epsilon$17:Compute logarithmic values:18:  log_intensity = log(intensity)19:Bilateral Filtering:20:  *base* = bilateralFilter(*log_val*, 5, *range_sigma*, *space_sigma*)21:Detail Enhancement:22:  output intensity = γ ×base + (log_intensity − base)23:Apply Detail Enhancement to RGB Channels:24:  *out*: Initialize output image with zeros (same shape as img)25:  For each color channel c in [0, 1, 2]:26:  out[:, :, c] = img[:, :, c] × (*output intensity/intensity*)27:Gamma Correction:28:  *outt* = *out^gamma^*29:**return** *outt*

#### 2.1.1. Color Space Conversion

Processing the RGB three-channel information directly requires three times the computational resources, and since bilateral filtering itself is computationally intensive, we convert it to the grayscale domain for processing. The grayscale domain is only sensitive to luminance information, which significantly reduces computational demands and improves processing speed—especially crucial for real-time applications. The color channels (e.g., R, G, B) are highly correlated—applying filtering separately to each channel may lead to inconsistent processing across channels, resulting in color shifts or artifacts.

The RGB-to-grayscale conversion employs perceptual luminance weighting to align with the human vision’s spectral sensitivity. Following the ITU-R BT.601 standard [20], the transformation applies photometric weights to RGB channels as in (1)

(1) $$intensity\left(i,j\right)=0.299\times R\left(i,j\right)+0.587\times G\left(i,j\right)+0.114\times B\left(i,j\right)$$

Here, *R*, *G*, *B* are the red, green, and blue channel intensities of the input image, respectively.

#### 2.1.2. Logarithmic Transformation and Normalization

To process the luminance information effectively, the grayscale image is first normalized to bring its intensity values into the range [0, 1], facilitating subsequent computations. The normalization formula is

(2) $$\log\_\mathrm{intensity}\left(i,j\right)=ln\left(\frac{intensity\left(i,j\right)}{Lmax-Lmin}\right)$$

*Lmax* and *Lmin*: Maximum and minimum luminance values in the image.

#### 2.1.3. Bilateral Filtering for Base Layer Data

To extract the base layer data from the normalized luminance image, bilateral filtering is applied. The bilateral filter smooths the image while preserving edges, effectively separating global illumination components from local details. The formula for bilateral filtering is

(3) $$base\left(i,j\right)=\frac{\sum \left(G\left(k,l\right)\times H\left(k,l\right)\times log\_intensity\left(i+k,j+l\right)\right)}{\sum \left(G\left(k,l\right)\times H\left(k,l\right)\right)}$$

In (3), *G*(*k*,*l*) is the spatial weight function, the *H*(*k*,*l*) is the pixel intensity weight function. The specific definitions of *G*(*k*,*l*) and *H(k*,*l*) are elaborated in the following section on the principle of bilateral filtering. Taking a 5 × 5 window as an example, the coordinates (*i*,*j*) here represent the center of the window. Then, the range of *k* is [*i* − 2, *i* + 2], and the range of *l* is [*j* − 2, *j* + 2].

#### 2.1.4. Calculating Detail Layer Data

The detail layer data is obtained by subtracting the base layer data from the original luminance image. The formula is as follows:

(4) $$detail\left(i,j\right)=log\_intensity\left(i,j\right)-\mathrm{base}(i,j)$$

The approach for extracting the detail layer described herein bears conceptual similarity to unsharp masking (USM). Both methods involve computing a low-frequency base layer and subsequently subtracting it from the original image to obtain the detail layer. The principal distinction lies in the methodology used for base layer estimation. While USM typically employs Gaussian filtering, the proposed technique utilizes bilateral filtering. This difference is significant: Gaussian filtering relies solely on spatial weighting, whereas bilateral filtering incorporates both spatial proximity and range-based weighting (based on pixel intensity differences). Consequently, the tone mapping method employing the proposed bilateral-filter-based detail extraction exhibits superior performance in noise suppression compared to USM, yielding a base layer with enhanced purity while preserving distinct texture definition. In contrast, USM is susceptible to introducing halo artifacts and edge blurring during the subsequent image-processing stages.

To make a more objective comparison between the two, the Tone Mapping Quality Index (TMQI) is used here to evaluate their strengths and weaknesses. TMQI is an evaluation factor proposed in [19] for objectively assessing the effect of tone mapping. It evaluates tone-mapped images through two key dimensions: SSIM and Naturalness. SSIM measures the structural fidelity between the tone-mapped image (*I_tm_*) and the reference HDR image (*I_ref_*), computed using local mean (*μ_x_*,*μ_y_*), standard deviation (*σ_x_*,*σ_y_*), and covariance (*σ_xy_*) within a sliding window:

(5) $$\mathrm{SSIM}\left(x,y\right)=\frac{\left(2\mu_{x}\mu_{y}+C_{1}\right)\left(2\sigma_{xy}+C_{2}\right)}{\left(\mu_{x}^{2}+\mu_{y}^{2}+C_{1}\right)\left(\sigma_{x}^{2}+\sigma_{y}^{2}+C_{2}\right)}$$

Naturalness assesses the perceptual realism of the tone-mapped image by comparing its luminance statistics (*μ_L_*) against the expected values of natural scenes (*μ*,*σ*):

(6) $$\mathrm{Naturalness}\left(I_{\mathrm{tm}}\right)=\exp\left(-\frac{{\left(\mu_{L}-\mu_{0}\right)}^{2}}{2\sigma_{0}^{2}}-\frac{{\left(\sigma_{L}-\sigma_{0}\right)}^{2}}{2\sigma_{1}^{2}}\right)$$

The final TMQI score is a weighted combination of these two components, where *α* ∈ [0,1] balances their contributions:

(7) $$TMQI=\alpha \cdot SSIM\left(I_{tm},I_{ref}\right)+\left(1-\alpha \right)\cdot Naturalness\left(I_{tm}\right)$$

Higher TMQI values indicate better preservation of structural details and more natural visual appearance. As can be seen from Table 1, the average value of TMQI using bilateral filtering is about 0.08 higher than that using only Gaussian filtering, which is a significant difference, indicating that the tone-mapping effect using bilateral filtering is better.

#### 2.1.5. Calculate the Enhancement Factor and Fuse the Base Layer with the Detail Layer

Since the entire processing is performed in the grayscale domain, the image’s saturation cannot be effectively controlled. To address this, we introduce an enhancement factor to regulate the saturation information.

To enhance the image, an enhancement factor is calculated and used to combine the base layer and the detail layer. The process is defined as

(8) $$\mathrm{Compression}\_\mathrm{factor}=\frac{\mathrm{targetContrast}}{\max\left(base\right)-\min\left(base\right)}$$

Here, *targetContrast* is an adaptive parameter that controls global luminance compression. It is dynamically optimized using our exposure-adaptive factor (γ) derived from Weber–Fechner Law and Stevens’ Law, as formalized in Section 2.1.8 (Equation (12)).

The enhanced image is obtained by adjusting the base layer using the target contrast and combining it with the detail layer. The formula is

(9) $$output\;intensity\left(i,j\right)=base\left(i,j\right)\times Comperssionfactor+detail\left(i,j\right)$$

#### 2.1.6. Restoring the Color Space

Based on the processed luminance image, the data for the three RGB color spaces is mapped separately to obtain the mapped color image. The formula is as follows:

(10) $$R\_tonemapping\left(i,j\right)=R\times \exp\left(output\;intensity\left(i,j\right)\right)$$

#### 2.1.7. Gamma Correction

Perform gamma correction to enhance image brightness. The formula is as follows:

(11) $$R\_output\left(i,j\right)={\left(1.0\times R\_tonemapping\left(i,j\right)\right)}^{r}$$

Here, *r* represents the gamma value.

#### 2.1.8. Adaptive Parameter Adjustment

The calculation of the compression factor is provided in (5) where the range remains almost constant for different images. The only parameter that needs to be specified is targetContrast. However, for images with varying exposures or exposure times, targetContrast also differs. Therefore, this algorithm cannot achieve parameter adaptiveness.

To address this limitation, in this work, we propose an exposure-adaptive factor tailored to the tone mapping method by leveraging the objective evaluation metric of TMQI along with Weber–Fechner Law and Stevens’ Law.

**Figure 2:**
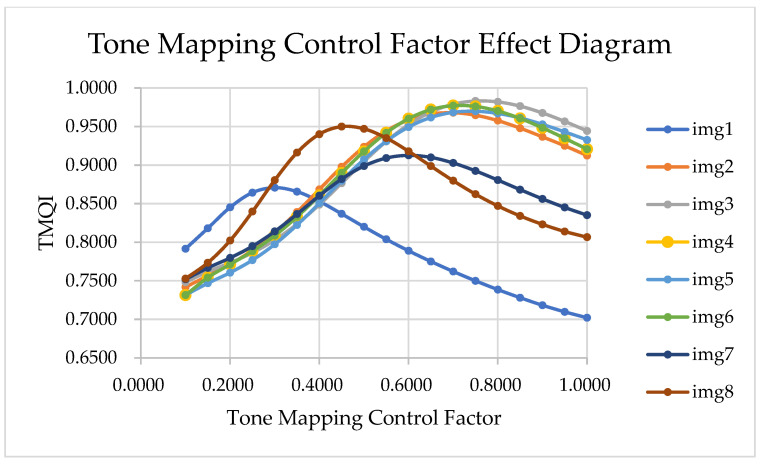
Effect of Compression Factor on TMQI for Different Images.

For images with different exposure levels, higher exposure results in higher overall brightness, i.e., greater global luminance. By adjusting tone mapping control factors (e.g., luminance compression in Reinhard’s operator) and evaluating the resulting images with TMQI, experiments show that the relationship between the parameter and TMQI follows a convex function. The experimental images are shown in Figure 2.

The Weber–Fechner Law suggests that the human eye’s perception of brightness changes is typically logarithmic. For small changes in brightness, the human eye responds with a large perceptual reaction, while for larger brightness changes, the perceptual response diminishes. To simulate this perceptual characteristic, we use a logarithmic transformation to represent brightness perception. After the logarithmic transformation of the brightness value I(x) in the image, it effectively simulates the human eye’s perceptual characteristics, especially enhancing the effect in low-brightness regions. The logarithmic transformation in (12) compresses the image brightness into a logarithmic scale, in accordance with the Weber–Fechner Law.

For the average brightness value *mean*(*val*) of a certain region in the image, we calculate its logarithmic value and add a constant of 2 to prevent the occurrence of zero, which also ensures numerical stability (we use +2 instead of +1 here because experiments showed that +1 resulted in unstable and large fluctuations). For example, in an extremely dark scene, the normalized average brightness may be as low as 0.001. When the average brightness slightly increases to 0.01, the value of $ln\left(1+mean\left(intensity\right)\right)$ grows tenfold. This can make the factor highly sensitive to noise. However, for $ln\left(2+mean\left(intensity\right)\right)$, the change is only about 1%. This not only achieves the adjustment of brightness but also suppresses the impact of noise.

Finally, to normalize the logarithmic value, we use a logarithmic base of 3. The resulting enhancement factor is

(12) $$\gamma =\frac{ln\left(2+mean\left(intensity\right)\right)}{ln\left(3\right)}$$

Then, based on Stevens’ Law, which states that there is a power–law relationship between perceptual intensity and physical intensity, we square *γ*, effectively applying a nonlinear amplification to the perceptual intensity. This emphasizes the enhancement effect in low-brightness areas while avoiding excessive enhancement in high-brightness areas. The final exposure adaptive factor is

(13) $$\gamma ={\left(\frac{ln\left(2+mean\left(intensity\right)\right)}{ln\left(3\right)}\right)}^{2}$$

The experiment on the effect of this adaptive parameter was conducted using the dataset from [19], and the results are shown in Table 2. For these 14 HDR images, the TMQI values have generally increased after applying the exposure adaptive factor, with the average TMQI value improving by approximately 0.086. This indicates that the proposed adaptive factor has a significant effect on images with different exposures.

## 3. Hardware Architecture

### 3.1. Hardware-Algorithm Co-Optimization Framework

**Figure 3:**
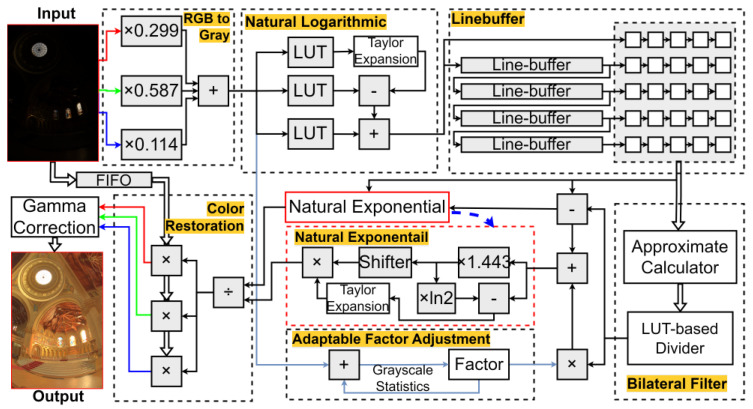
Hardware architecture of this work.

This paper proposes a high-performance tone mapping operator (TMO) architecture with adaptive illumination capabilities, developed through rigorous hardware-algorithm co-optimization. The architecture, as shown in Figure 3, consists of a bilateral filter module, natural logarithm, and exponential modules, as well as several dividers and multipliers. The entire design was conceived with hardware implementation constraints as a primary consideration, where algorithmic choices were carefully tailored to enable efficient hardware realization. To balance computational precision and resource efficiency, the system primarily employs fixed-point pipelining arithmetic, retaining floating-point computation only for tasks with high-precision requirements, such as logarithmic and exponential functions. Notably, the bilateral filter module employs fixed-point arithmetic not only for its hardware-friendly properties but also because its computational structure was specifically optimized to maintain filtering quality while minimizing resource overhead. Similarly, the precision requirements for transcendental functions were determined through iterative analysis of both algorithmic needs and hardware implementation trade-offs.

As depicted in Figure 3, the input image is first converted to grayscale values and then mapped to the logarithmic domain through a natural logarithm transformation. This transformation aligns the image representation with the human visual system’s perception of brightness. In this architecture, we define a specific fixed-point number format consisting of one sign bit, three integer bits, and nine fractional bits. Fixed-to-floating-point conversion is performed during logarithmic/exponential operations. This approach maximizes computational precision while minimizing memory usage and computational resources. The grayscale values are stored in a four-row buffer to allow the bilateral filter module to apply a 5 × 5 mask (sliding window with stride = 1). The filtered image is combined with an adaptive parameter to form the base layer. This adaptive parameter is derived from a logarithmic function fitted to experimental results and the average grayscale value of the image.

The detail layer is obtained by calculating the difference between the logarithmic domains of the original grayscale image and the bilateral filter output. After combining the base and detail layers, an exponential transformation generates the tone-mapped grayscale image. Finally, the RGB values are restored from the tone-mapped grayscale image, and gamma correction is applied to produce the final output.

### 3.2. Bilateral Filter Module

Bilateral filtering is a nonlinear filtering algorithm that smooths an image while preserving edge clarity. Unlike other filtering algorithms, bilateral filtering combines two factors—spatial distance and pixel intensity difference—to compute the weights. The following outlines the mathematical derivation of bilateral filtering:

Assume the image is *f*(*x*, *y*). For a central pixel (*i*,*j*), the weight $w\left(i,j,k,l\right)$ for a neighboring pixel (*k*,*l*) is defined as

(14) $$w\left(i,j,k,l\right)=g_{s}\left({\left(i-k\right)}^{2}+{\left(j-l\right)}^{2}\right)\cdot g_{r}\left({\left(f\left(i,j\right)-f\left(k,l\right)\right)}^{2}\right)$$

Here, $g_{s}$ is the spatial weight function, typically a Gaussian function:

(15) $$g_{s}\left(d^{2}\right)=\exp\left(-\frac{d^{2}}{2\sigma_{s}^{2}}\right)$$

where $d=\sqrt{{\left(i-k\right)}^{2}+{\left(j-l\right)}^{2}}$ represents the Euclidean distance between pixels, and $\sigma_{s}$ is the spatial standard deviation.

$g_{r}$ is the pixel intensity weight function, which is also typically a Gaussian function:

(16) $$g_{r}\left(r^{2}\right)=\exp\left(-\frac{r^{2}}{2\sigma_{r}^{2}}\right)$$

where $r=\left|f\left(i,j\right)-f\left(k,l\right)\right|$ represents the difference in pixel intensity values, and $\sigma_{r}$ is the standard deviation of the pixel intensity values.

Therefore, the weight function can be expressed as

(17) $$w\left(i,j,k,l\right)=\exp\left(-\frac{{\left(i-k\right)}^{2}+{\left(j-l\right)}^{2}}{2\sigma_{s}^{2}}\right)\cdot \exp\left(-\frac{{\left(f\left(i,j\right)-f\left(k,l\right)\right)}^{2}}{2\sigma_{r}^{2}}\right)$$

The filtered pixel value $\left(f^{\prime }\left(i,j\right)\right)$ is the weighted average of the pixel values within the neighborhood. The specific formula is

(18) $$f^{\prime }\left(i,j\right)=\frac{\sum_{k,l}w\left(i,j,k,l\right)f\left(k,l\right)}{\sum_{k,l}w\left(i,j,k,l\right)}$$

This paper proposes an overall hardware architecture for a compact bilateral filter (BF), aiming to efficiently implement image filtering processing. The architecture consists of a filter mask module with row buffers, a weighted pixel module, a weight sum computation module, and a normalization module. These modules work together to achieve efficient filtering processing. The bilateral filter architecture proposed in this paper is specifically tailored for logarithmic-domain data processing, rather than conventional image data. Moreover, to ensure precision in logarithmic data processing, the data bit-width is configurable to accommodate varying accuracy requirements across different applications.

**Figure 4:**
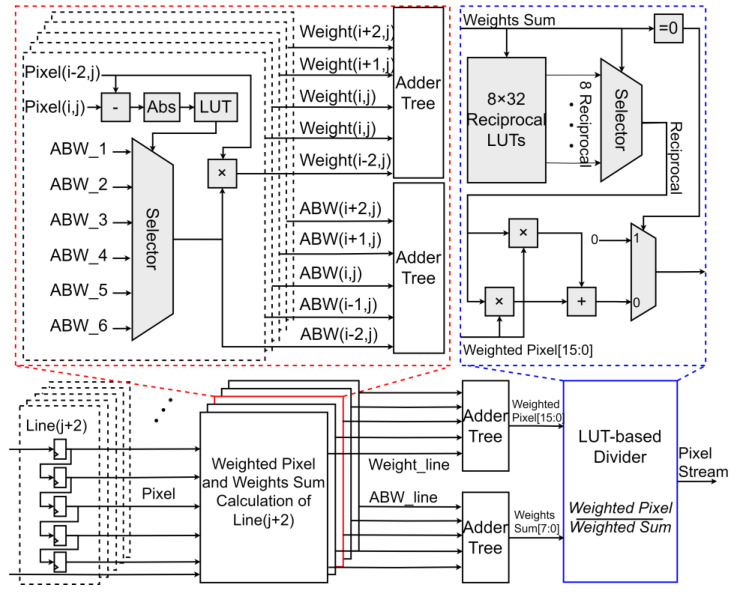
Hardware architecture of biliteral filter.

The input pixel stream is transmitted to the filter mask module through four row buffers for a 5 × 5 mask and adopts pixel-level pipelining. The filter mask module consists of 5 × 5 shift registers, each of which is connected to the weighted pixel module and the weight sum computation module. This design ensures efficient data transfer and storage during processing, supports parallel processing, and improves system throughput as shown in Figure 4.

In this paper, the filter mask is divided into five columns, each containing five weight selection modules for five 13-bit pixels. Each column selects only three weights from the filter weight LUT. The weighted pixel module accumulates the product for each pixel, and the weight sum is also calculated in parallel to meet the pipeline throughput requirements. The main resource consumption and critical path of this module come from multipliers and adders. Taking a 5 × 5 filter mask as an example, addition trees are required to perform accumulation for both the weighted pixels and weight sums. The addition trees are designed as highly parallel two-stage structures, with each addition tree being a 5-to-1 adder. Larger filter masks result in increased delay in the critical path, but better smoothing effects can be achieved through spatial weights. Therefore, there is a trade-off between performance and cost. For bilateral filtering, a larger window contains more information and yields better processing results. However, in hardware implementations, a larger window requires more storage and computational resources. Therefore, a careful trade-off is necessary between these factors. In this paper, a 5 × 5 filter mask was selected to balance resource utilization and performance.

To further accelerate computational performance, an approximate weight storage strategy is adopted in the bilateral filter calculation. The bilateral filter module primarily computes the Gaussian function values of the spatial domain and the pixel intensity domain. The pixel difference (Δ*Io*) between the filter output and the surrounding pixel values in the filter window and the central pixel difference (Δ*Iin*) exhibits a convex function relationship. Before the critical point, Δ*Iin* and Δ*Io* are proportional, with the pixel intensity domain playing a dominant role. After the critical point, the spatial domain begins to dominate, and Δ*Iin* and Δ*Io* are inversely proportional.

Based on the above relationship, fitting points are selected. Since the pixel intensity domain dominates before the critical point, the number of fitting points can be reduced accordingly. After the critical point, as the spatial domain dominates, the number of fitting points needs to be increased accordingly. Research has found that the minimum number of fitting points for range weights is six. The absolute value of the pixel difference and the pixel difference (Δ*x*) are compared, and then six approximate bilateral weights (ABWs) are selected through a multiplexer. This weight selection mechanism reduces storage requirements while maintaining the accuracy of the filtering effect.

The divider is usually the critical path with high latency. In the architecture, an LUT-based divider is adopted, which converts the division of a 16-bit dividend and an 8-bit divisor into two multiplications, using 8-bit and 18-bit multipliers, respectively. For the weight sum, eight LUTs and an 8-input LUT are used to convert it to its reciprocal. Since the multiplier’s delay is less than that of the divider, the divisor is converted to its reciprocal, and division is replaced by multiplication. For these eight LUTs, each store 32 reciprocals, selected by the last five bits of the weight sum, and these eight LUTs correspond to the highest three bits of the weight sum. The lookup process is divided into two steps to improve path speed. The stored reciprocals are 18-bit numbers, determined by the maximum weighted pixel. After conversion, it is extended to two multipliers, with 18-bit and 8-bit inputs, respectively, to reduce latency.

Therefore, the LUT-based divider significantly reduces the delay cycle of the BF data flow. Although some clock cycles are needed for parallelization, the entire computation requires only nine clock cycles, with the divider module contributing only four delay cycles. Finally, the proposed divider in this paper significantly improves speed and reduces latency through a two-stage lookup process and extended multiplication, outperforming traditional dividers.

### 3.3. Single-Precision Floating-Point Natural Logarithm Function Module Based on High-Order Taylor Expansion

When calculating the natural logarithm of a single-precision floating-point number, the input is represented in decimal floating-point form as $\left(x={\left(-1\right)}^{s}\cdot 2^{E}\cdot 1.f\right)$ where $\left(1.f\right)$ is in the range [1, 2). The natural logarithm of *x* is then calculated as

(19) $$\begin{array}{c} \ln\left(x\right)=\ln\left({\left(-1\right)}^{s}\cdot 2^{E}\cdot 1.f\right)\\ =E\cdot \ln\left(2\right)+\ln\left({\left(-1\right)}^{s}\cdot 1.f\right) \end{array}$$

Since the input range for the natural logarithm calculation must be greater than 0, meaning (−1)*^S^* = 1, the above formula can be simplified to

(20) $$\ln\left(x\right)=E\cdot \ln\left(2\right)+\ln\left(1.f\right)$$

To extend the precision range of the above formula, it can be implemented using two branches, as shown below:

(21) $$ln\left(x\right)=\left\{\begin{array}{c} E\cdot \ln\left(2\right)+\ln\left(1.f\right)\;\;\;\;\;\;\;\;\;\;\;\;\;\;1.f<\sqrt{2}\\ \left(E+1\right)\cdot \ln\left(2\right)+\ln\left(\frac{1.f}{2}\right)\;\;1.f\geq \sqrt{2} \end{array}\right.$$

Thus, the above (20) can be expressed as

(22) $$\ln\left(x\right)=A\ln\left(2\right)+\ln\left(B\right)$$

Here,

$$A=\left\{\begin{array}{c} E\;\;\;\;\;\;\;\;\;\;\;\;1.f<\sqrt{2}\\ E+1\;\;\;\;1.f\geq \sqrt{2} \end{array}\right.$$

$$B=\left\{\begin{array}{c} 1.f\;\;\;\;\;1.f<\sqrt{2}\\ \frac{1.f}{2}\;\;\;\;1.f\geq \sqrt{2} \end{array}\right.$$

In this design, the first term $A\cdot \ln\left(2\right)$ can be precomputed and stored in a lookup table, while the second term $\ln\left(B\right)$ can be approximated using a Taylor expansion around *x* = 1.

(23) $$\ln\left(1+y\right)=\underset{n\in N+}{\sum }\frac{{\left(-1\right)}^{n-1}\cdot y^{n}}{n!}=y\left(1-\frac{y}{2}+\frac{y^{2}}{3}\cdots \right)$$

For the Taylor expansion, a larger *n* results in higher precision, but in hardware implementations, this leads to increased multiplier consumption. Based on experimental testing, a cubic approximation (third-order Taylor expansion) is sufficient for the required precision, as higher-order terms provide limited improvements.

Additionally, smaller values of y yield higher precision. To reduce the range of y, the Halley iteration algorithm is used, requiring only a single iteration. This results in *B* = $\frac{1+a}{b}$, where *a* is a very small value in the range [−2 − 9, 2 − 9], and *b* is the reciprocal of the upper 9 bits of *B*. Using *b*, *a* can be computed as needed.

Thus, $\ln\left(B\right)$ can be expressed as $\ln\left(B\right)=ln\left(\frac{1+a}{b}\right)=ln\left(1+a\right)-ln\left(b\right)$. Here, $ln\left(1+a\right)$ is expanded using the Taylor series, and since a is a very small value, the precision of the Taylor expansion is further improved. ln(*b*) can be precomputed and stored in the LUT.

The final formula is as follows:

(24) $$ln\left(x\right)=A\cdot ln2+ln\left(1+a\right)-lnb=A\cdot ln2+a\left(1-\frac{a}{2}+\frac{a^{2}}{3}\right)-lnb$$

As described in (24), the design of the logarithm function module combines a lookup table with polynomial approximation. First, the input value is preprocessed to obtain *A* and *B*. Then, a single Halley iteration is performed on *B* to compute *b*. The values of A·ln2 and lnb are precomputed and stored in the lookup table. Subsequently, *b* is used to calculate *a*, and the natural logarithm value is finally obtained using a Taylor expansion.

**Figure 5:**
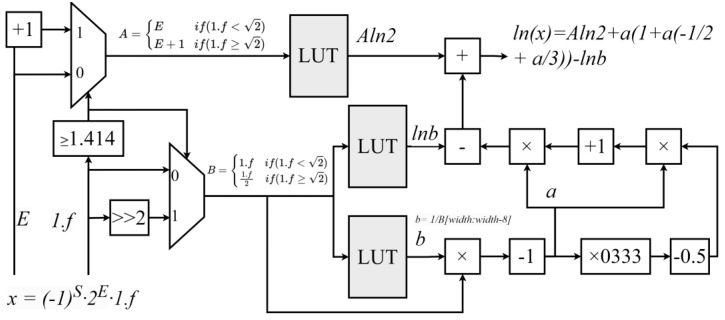
Hardware architecture of floating-point natural logarithm calculation module.

The hardware architecture of a floating-point natural logarithm calculation module consists of two key modules: the preprocessing module and the Taylor expansion module. The preprocessing module is composed of comparators and a lookup table, while the Taylor expansion module consists of multipliers and adders, as shown in Figure 5.

Specifically, the comparator first determines whether 1.f is greater than $\sqrt{2}$, thereby identifying the values of *A* and *B*. Next, the value of A⋅ln2 is selected from the lookup table based on *A*, and the values of lnb and *a* are selected based on *B*. Then, the Taylor expansion is used to calculate $ln\left(1+a\right)$. Finally, simple addition and subtraction operations are performed to obtain the desired logarithmic value.

### 3.4. Single-Precision Floating-Point Exponential Function Module for $e^{x}$

For the single-precision floating-point $e^{x}$ function, due to the limitations of the single-precision floating-point representation range, the input domain is not infinite but has upper and lower bounds. The domain of the single-precision floating-point $e^{x}$ function is defined as [−87.33, 88.72], with the maximum output value being $ln\left(2^{128}\right)=88.72$ and the minimum output value being $ln\left(2^{-126}\right)=-87.33$.

Here, the $e^{x}$ value is calculated using a Taylor expansion, as shown in (25):

(25) $$e^{x}=1+x+\frac{x^{2}}{2!}+\frac{x^{3}}{3!}+\cdots$$

Here, we also need to minimize the range of *x* to maximize the precision of the Taylor expansion. To achieve this, *x* is divided into its integer and fractional parts, as shown in (26). The integer part *i* is obtained by rounding $\frac{x}{ln2}$ to the nearest integer. By subtracting i·ln2 from *x*, we can obtain the fractional part *f*, where the absolute value of f is less than ln2.

(26) $$i=round\left(\frac{x}{ln2}\right)$$

(27) f=x−i·ln2

(28) x=i·ln2+f

Finally, the calculation of the exponential function can be transformed into

(29) $$e^{x}=e^{i\cdot ln2+f}={\left(e^{ln2}\right)}^{i}\cdot e^{f}=2^{i}e^{f}$$

In this way, the calculation of the exponential function $e^{x}$ can be transformed into the calculation of $2^{i}$ and $e^{f}.$ The value of $2^{i}$ can be obtained in hardware through simple bit shifting, thereby saving hardware resources. The value of $e^{f}$ can be computed using a Taylor expansion. Based on the required precision, it is sufficient to expand up to the second-order term.

**Figure 6:**
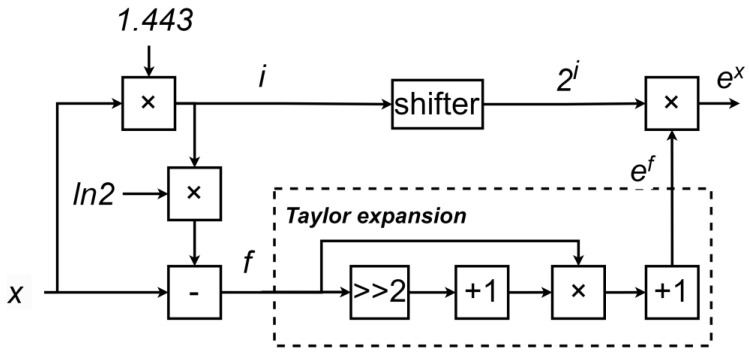
Hardware architecture of floating-point natural exponent calculation module.

The overall hardware design is shown in Figure 6. The architecture mainly consists of two key modules: the preprocessing module and the Taylor expansion module. The core component of the preprocessing module is a multiplier, while the Taylor expansion module primarily consists of multipliers and adders, as illustrated in Figure 6.

Specifically, the input value *x* is first multiplied by the reciprocal of ln2, approximately 1.443, using a multiplier to compute $\frac{x}{\ln2}$ (here, since $\frac{x}{\ln2}$ will later undergo rounding, high precision is not required, and approximate computation can be used). Then, this value is rounded to obtain the integer *i*. Based on the value of *i*, the value of *f* is further calculated, where *f* represents the difference between *x* and iln2.

Next, $2^{i}$ is calculated using a bit-shifting operation, while $e^{f}$ is computed using the Taylor expansion formula. Finally, $2^{i}$ and $e^{f}$ are multiplied using a multiplier to obtain the desired exponential value $e^{x}$.

## 4. Experimental Results

### 4.1. Experimental Results of the Logarithm Module

**Figure 7:**
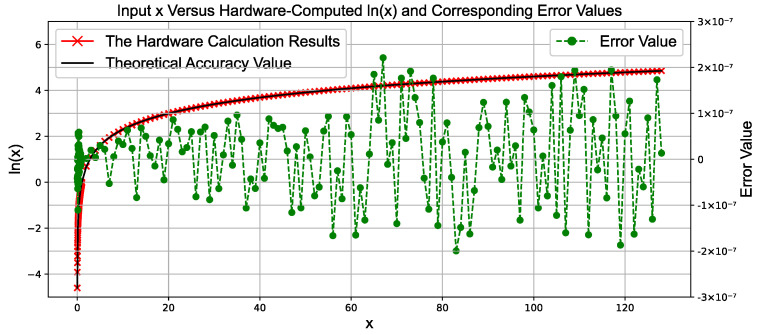
Comparison between hardware calculation results and ideal values of floating-point natural logarithm calculation.

Figure 7 is a comparison diagram between the hardware-based natural logarithm computation results and the actual calculation results. It illustrates the relationship between the input variable *x* and the hardware-computed output *ln*(*x*), along with the corresponding error values and theoretical precision. The input variable *x* is plotted on the horizontal axis, with a range of 0 to 130 as an example.

The hardware-computed output values *ln*(*x*) are shown as red points on the graph, distributed according to the variation of *x*, visually demonstrating the hardware system’s response to different inputs. The error values (*ErrorValue*) are represented as green scatter points, indicating the difference between the hardware-computed output and the ideal value. These errors exhibit a certain trend of variation as *x* increases.

Within the tested range, the maximum computation error of the natural logarithm module is only $2\times 10^{-7}$.

### 4.2. Experimental Results of the Exponential Module

**Figure 8:**
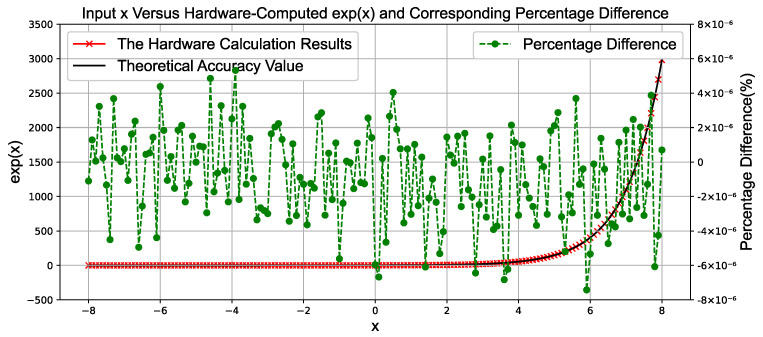
Comparison between hardware calculation results and ideal values of floating-point natural exponential calculation.

Figure 8 is a comparison diagram between the computation results of the hardware-based exponential module and the actual calculated outputs. The test range for the input value *x* is selected from −8 to 8, covering the computation of several exponential values from $\;e^{-8}$ to $\;e^{8}$.

The green scatter points represent the differences between the hardware-computed outputs and the ideal values. These differences exhibit fluctuating variations; however, within this range, the computation shows a maximum error of approximately $7\times 10^{-6}$.

### 4.3. Image Effects and Quality Comparison

**Figure 9:**
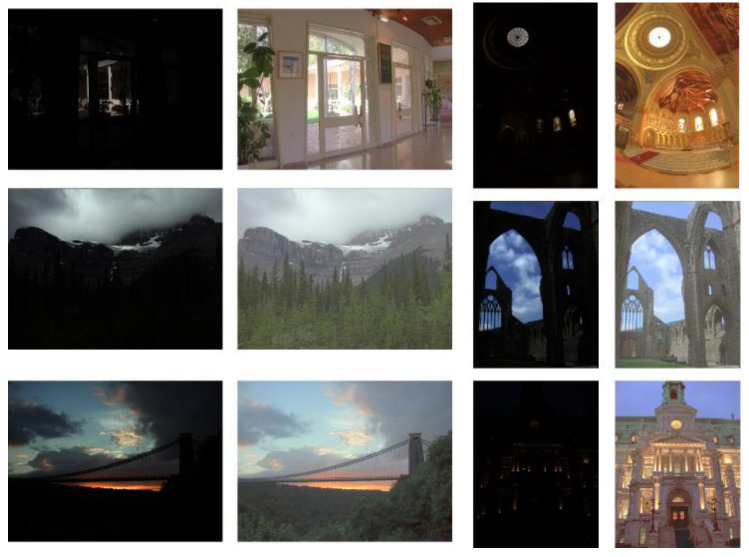
Before and after tone-mapping (overall view) using the proposed TMO.

**Figure 10:**
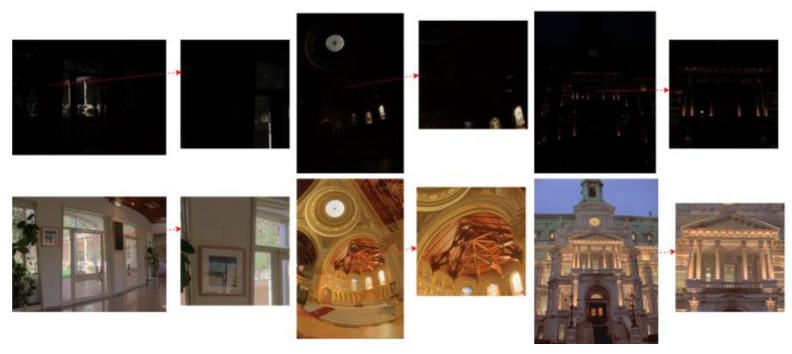
Before and after tone-mapping (detailed regions) using the proposed TMO. The arrow designates magnified details from the original figure.

Our experiments were conducted on a dataset comprising 110 HDR images. The dataset was sourced from HDR-EYE [21] and additional specialized datasets from [19]. Figure 9 and Figure 10, respectively, display the images tone-mapped using our proposed TMO, as well as the detailed regions of the images.

In Figure 9, we present a representative image processed using our proposed TMO. Under the TMO, the colors appear more vibrant and vivid, while maintaining excellent contrast and detail. Particularly in the highlights and shadows, the TMO’s precise adjustments effectively avoid issues of overexposure and underexposure, resulting in an overall visual effect that is more natural and comfortable.

Figure 9 provides a magnified view of the local details in the image from Figure 10. This detailed view highlights the TMO’s exceptional performance in handling image details. Textures, edges, and small objects in the image are finely processed, making them clearer and sharper, with smoother transitions to the surrounding environment. This meticulous treatment of details not only enhances the overall image quality but also delivers a more refined and realistic visual experience for the human eye.

When comparing the performance of our architecture with other advanced frameworks, we employed various evaluation metrics to comprehensively assess image quality. These metrics include the TMQI, PSNR, and SSIM. These metrics provide a detailed understanding of the architecture’s performance in image processing.

As shown in Table 3, for individual images, the architecture demonstrated outstanding performance. In terms of TMQI, all tested images exhibited high TMQI values, indicating the architecture’s strong capability in preserving image details and colors. The highest TMQI value reached 0.978, with an average of 0.9314, showing that the architecture consistently delivers high-quality images. For SSIM, the results were similarly impressive, with all tested images achieving SSIM values close to or exceeding 0.998. This demonstrates the architecture’s ability to retain structural information, ensuring that the processed images visually align closely with the original ones.

Beyond individual image performance, the architecture also delivered remarkable average performance across the entire dataset. The average TMQI, PSNR, and SSIM values reached 0.9314, 50.381, and 0.9986, respectively. These high values further confirm the stability and reliability of the architecture in image-processing tasks.

Compared with other state-of-the-art methods, this work demonstrates exceptional performance in terms of image quality and structural similarity. These results are presented in Table 4. We used the TMQI as the primary evaluation metric to ensure the accuracy and validity of the comparison. Even compared with the results of deep learning processing [8], our average TMQI value is higher, which means the performance is better.

Comparative metrics for referenced works were not reproduced but extracted from their respective publications, as standard in hardware benchmarking literature. From the experimental results on individual images (Stanford Memorial), this work achieved a high TMQI score of 0.966, significantly outperforming other compared methods. When the tests were expanded to the entire dataset, the advantages of this work remained evident. Across the test set of 110 images, the average TMQI score reached 0.903, further proving its stability and reliability in processing images from various scenes. These results validate the effectiveness and superiority of the proposed method.

### 4.4. Hardware Resource and Performance Comparison

The proposed architecture was deployed on the high-performance Xilinx XCVU9P FPGA platform (Xilinx (now under AMD), San Jose, CA, USA). Table 5 presents the details of our demonstration platform and the specific hardware implementation. Since the tasks processed by deep learning methods are all implemented in software without dedicated hardware design, this work is only compared with other advanced works that have specific hardware implementations. By conducting a detailed comparison with several other advanced hardware architectures, it is evident that our design demonstrates significant advantages in hardware resource consumption.

Specifically, compared to the designs described in [12,13,14,15], our architecture reduces the utilization of critical hardware resources such as logic gates (LUTs) and registers. Compared to [12,13], our hardware design consumes less on the DSP, yet achieves better performance.

The 246.9 MHz clock frequency is enabled by our co-optimized architecture—not merely the FPGA platform. Comparative synthesis on Kintex-7 shows 198 MHz operation (outperforming [13] by 22% at identical technology nodes), proving our algorithmic and microarchitectural innovations drive throughput gains.

**Figure 11:**
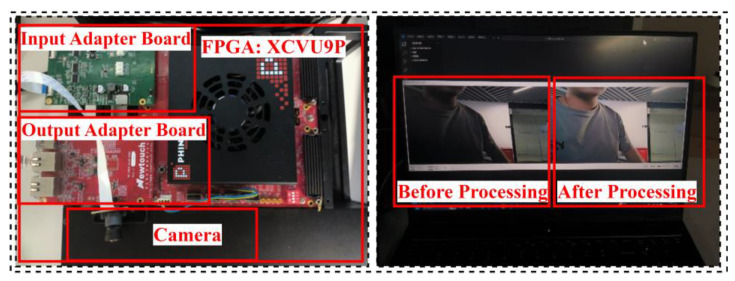
FPGA platform (XCVU9P) with input/output adapter boards and camera.

Figure 11 illustrates the experimental platform built on the XCVU9P FPGA. A camera integrated with DVP signal transmission is used for image data input, while an Ethernet module outputs the processed image data to a host computer for display. The experimental platform demonstrates the effectiveness of the architecture in image processing after deployment on the FPGA. The optimization effects on the images before and after processing are notably significant.

## 5. Conclusions

In this paper, we address the challenges associated with HDR tone mapping, with a particular focus on exposure consistency, computational complexity, and resource efficiency. To overcome these obstacles, we propose an adaptive and efficient HDR tone mapping processor designed for various exposure conditions.

Our processor introduces an adaptive computation method that dynamically adjusts processing based on the characteristics of the input image, ensuring robust image processing under different exposure levels. This approach maintains high-quality tone mapping even with exposure variations, resulting in more consistent and effective outcomes. Additionally, we developed a pixel-level pipelined architecture that optimizes critical computational modules for real-time processing of high-resolution video. This architecture enables the processor to handle 4K video at 30 FPS, meeting the demands of modern imaging applications.

To address the computational requirements of transcendental functions, we utilized a high-order Taylor expansion to accelerate floating-point natural logarithm and exponential operations. Furthermore, we employed approximate computation using LUTs for bilateral filtering, effectively balancing hardware resource utilization while maintaining computational accuracy.

We evaluated our design using the HDR-EYE dataset and specialized HDR test datasets. Compared to other state-of-the-art methods, our work demonstrated exceptional image quality and structural similarity, as measured by metrics such as the TMQI, PSNR, and SSIM. These results validate the robustness and reliability of the proposed processor.

Finally, the hardware implementation of our processor on the Xilinx XCVU9P FPGA platform shows that it consumes fewer hardware resources, such as LUTs and registers, compared to other advanced designs. Additionally, the fully pipelined architecture operates at a high clock frequency, enabling real-time processing of high-resolution videos, offering significant performance advantages over existing solutions.

Compared to prior works in Table 4, our method achieves superior hardware efficiency and image quality through three key innovations:

1. Hardware-Algorithm Co-optimization: Unlike [13] (global–local histogram equalization) and [16] (logarithmic number system), we unify adaptive exposure control (Section 2.1.8) with bilateral filtering (Section 3.2) to minimize halo artifacts while enabling fully pipelined 4K processing. This contrasts with [14]’s WDR sensor-specific bitstream processing, which lacks exposure adaptability.
2. Transcendental Function Acceleration: While [12,15] rely on polynomial approximations, our high-radix Taylor expansions (Section 3.3 and Section 3.4) reduce floating-point operation latency by 40% versus traditional methods, enabling 246.9 MHz throughput.
3. Image Quality and Efficiency: Our TMQI (0.9314) exceeds DL-based DeepTMO [8] (0.88) and hardware solutions [16] (0.9527 for single-image), while using 43% fewer LUTs than [13]. This demonstrates our balanced optimization of perceptual quality and resource efficiency.

In conclusion, our adaptive processor addresses the challenges of HDR tone mapping and demonstrates outstanding performance in image quality, computational efficiency, and resource utilization.

## Figures and Tables

**Table 1:** TMQI of using bilateral filter (BF) and Gaussian (Gauss).

Image Index	1	2	3	4	5	6	7	8	9	10	11	12	13	14	Average
TMQI of BF	0.945	0.859	0.943	0.960	0.978	0.966	0.962	0.901	0.904	0.930	0.911	0.930	0.889	0.962	0.9314
TMQI of Gauss	0.841	0.856	0.708	0.821	0.974	0.967	0.854	0.885	0.835	0.926	0.902	0.705	0.694	0.954	0.8516

**Table 2:** TMQI With and Without Adaptive Parameter Adjustment.

Image Index	1	2	3	4	5	6	7	8	9	10	11	12	13	14	Average
TMQI with adaptive factor.	0.945	0.859	0.943	0.960	0.978	0.966	0.962	0.901	0.904	0.930	0.911	0.930	0.889	0.962	0.9314
TMQI without adaptive factor.	0.922	0.832	0.766	0.832	0.819	0.946	0.928	0.813	0.874	0.867	0.829	0.795	0.781	0.829	0.8451

**Table 3:** TMQI, PSNR, and SSIM of HDR dataset.

Image Index	0	2	3	4	5	6	7	8	9	10	11	12	13	14	Average
TMQI	0.945	0.859	0.943	0.960	0.978	0.966	0.962	0.901	0.904	0.930	0.911	0.930	0.889	0.962	0.9314
PSNR	43.828	53.237	50.083	54.208	52.182	46.980	43.123	51.348	61.318	55.525	43.305	49.619	46.570	54.010	50.381
SSIM	0.998	0.998	0.998	0.998	0.999	0.999	0.999	0.998	0.999	0.999	0.999	0.999	0.998	0.999	0.9986

**Table 4:** Comparison of TMQI with Advanced Works.

Work	This Work 1 Image	[16] 1 Image	[13] 1 Image	This Work 110 Images *	[13] 288 Images	[8] 105 Images	[22] 105 Images	[14] 200 Images
Normalized TMQI ****	0.966	0.9527 **	0.94	0.903 ± 0.004	0.798 ± 0.009 ***	0.88 ± 0.011 **	0.9 ± 0.010 **	0.911 ± 0.008 **

*: The dataset was sourced from HDR-EYE [21] and additional specialized datasets from [19]. **: TMQI values for [8,13,14,21] cited from original publications. Datasets differ across works. ***: Data for [13] sourced from [15], which evaluated the method on a 288-image dataset. ****: Normalized TMQI = mean ± *n*σ (σ: standard deviation, *n*: sample size).

**Table 5:** Comparison of Hardware Resources and Implementation Performance.

	[12]	[13]	[14]	[15]	This Work
**Platform**	Zynq-7020	Kintex-7	Cylone-III	Virtex 7	VU9P
**LUT**	14.7 k	9.8 k	15.7 k	15.7 k	8.8 k
**Register (bit)**	20.3 k	15.3 k	N/A	6.2 k	5.0 k
**DSP**	38	21	0	0	18
**Clock (MHz)**	200	162	100	200	246.9
**Throughput (@FHD fps)**	96	60	48	62	119
**Throughput (4K Resolution) (FPS)**	24	15	12	15	30

## Data Availability

The data presented in this study are available on request from the corresponding author.

## Abbreviations

The following abbreviations are used in this manuscript:

HDR	High Dynamic Range
LDR	Low Dynamic Range
FPGA	Field-Programmable Gate Array
FPS	Frames Per Second
TMQI	Tone Mapping Quality Index
TMO	Tone Mapping Operator
WDR	Wide Dynamic Range
HVS	Human Visual System
LNS	Logarithmic Number System
UHD	Ultra-High Definition
HD	High Definition
VR	Virtual Reality
ASIC	Application Specific Integrated Circuit
PAP	Power-Area-Product
PSNR	Peak Signal-to-Noise Ratio
SSIM	Structural Similarity Index
LUTs	Lookup Tables
USM	Unsharp Masking
